# Mitochondrial DNA Instability Is Common in HIV-Exposed Uninfected Newborns

**DOI:** 10.3390/jcm10112399

**Published:** 2021-05-28

**Authors:** Audrey Monnin, Valérie Desquiret-Dumas, Nicolas Méda, David Goudenège, Céline Bris, Chipepo Kankasa, Mandisa Singata-Madliki, Thorkild Tylleskar, Vincent Procaccio, Nicolas Nagot, Philippe Van de Perre, Pascal Reynier, Jean-Pierre Molès

**Affiliations:** 1 Pathogenesis and Control of Chronic and Emerging Infections, INSERM, Etablissement Français du Sang, University of Montpellier, University of the Antilles, 34393 Montpellier, France; audrey.monnin@inserm.fr (A.M.); n-nagot@chu-montpellier.fr (N.N.); p-van_de_perre@chu-montpellier.fr (P.V.d.P.); 2 Department of Biochemistry and Genetics, University Hospital of Angers, 49933 Angers, France; VaDesquiret@chu-angers.fr (V.D.-D.); David.Goudenege@chu-angers.fr (D.G.); CeBris@chu-angers.fr (C.B.); viprocaccio@chu-angers.fr (V.P.); pascal.reynier@univ-angers.fr (P.R.); 3 UMR MITOVASC, CNRS 6015, INSERM U1083, University of Angers, 49933 Angers, France; 4 Centre MURAZ, Bobo-Dioulasso P.O. Box 390, Burkina Faso; nicolas.meda@gmail.com; 5 Department of Paediatrics and Child Health, University Teaching Hospital, Lusaka P.O. Box 50110, Zambia; ckankasa@zamnet.zm; 6 Effective Care Research Unit, University of Fort Hare, East London 5201, South Africa; mandisa.singata@gmail.com; 7 Centre for International Health, University of Bergen, 5020 Bergen, Norway; thorkild.tylleskar@cih.uib.no

**Keywords:** pediatric, HIV, mitochondria, genomic alterations, ARV, birth

## Abstract

Worldwide, one million HIV-exposed uninfected (HEU) children are born yearly, and chronic health impairments have been reported in these children. Mitochondrial DNA (mtDNA) instability and altered mtDNA content have been evidenced in these children, but an exhaustive characterization of altered mitochondrial genomes has never been reported. We applied deep mtDNA sequencing coupled to the deletion identification algorithm eKLIPse to the blood of HEU neonates (*n* = 32), which was compared with healthy controls (*n* = 15). Dried blood spots (DBS) from African HEU children were collected seven days after birth between November 2009 and May 2012. DBS from French healthy controls were collected at birth (or <3 days of life) in 2012 and in 2019. In contrast to the absence of mtDNA instability observed at the nucleotide level, we identified significant amounts of heteroplasmic mtDNA deletions in 75% of HEU children and in none of controls. The heteroplasmy rate of the 62 mtDNA deletions identified varied from 0.01% to up to 50%, the highest rates being broadly compatible with bioenergetic defect and clinical expression. mtDNA integrity is commonly affected in HEU neonates. The nature of the deletions suggests a mechanism related to aging or tumor-associated mtDNA instability. This child population may be at risk of additional mtDNA genetic alterations considering that they will be exposed to other mitotoxic drugs including antiretroviral or anti-tuberculosis treatment.

## 1. Introduction

Each year, almost one million children are born to HIV-infected mothers but are uninfected themselves (HIV-Exposed Uninfected (HEU) children) [1]. Recent studies clearly demonstrate that these children have an increased rate of mortality/morbidity [2,3,4], an increased rate of infectious diseases during infancy [5], and that they may suffer from health and developmental impairments including growth [6,7,8,9,10] and neurodevelopmental delay [6,7], altered immune response [11,12,13], and metabolic disorders [14,15,16]. The factors responsible for these health impairments are not fully understood, but it has been suggested that they result from antiretroviral (ARV) and/or HIV exposure(s), both during intra uterine life and breastfeeding.

ARVs, and particularly nucleoside reverse transcriptase inhibitors (NRTI), are well known for their mitochondrial genotoxicity due to the inhibition of the replication of the mitochondrial DNA (mtDNA) [17,18]. Physiologically, mtDNA has a higher rate of point mutations and rearrangements than nuclear DNA, due to the lack of histone scaffolding, a reduced efficacy of mtDNA repair mechanisms and a lower processivity of the DNA polymerase γ as compared to other cellular DNA polymerases [19,20]. In addition, its proximity with the reactive oxygen species (ROS), produced by respiratory chain, increases mtDNA instability. In the HIV context, this ROS production is enhanced by NRTI [21], chronic inflammation, and HIV-free proteins such as Gp120, Tat, Nef, and Vpr [22].

Among HEU children, mitochondrial genotoxicity has been investigated for many years, and studies mostly focus on the number of mtDNA copies per cell. In short, mtDNA content has been found to be lower in HEU neonates at birth compared to those born to HIV-uninfected mothers but higher than in neonates infected with HIV during pregnancy. Nonetheless, mtDNA defects in HEU children tend to normalize with age if drug or HIV exposure stops [23,24,25]. mtDNA point mutations and deletions persisting for years after birth have also been reported [26,27], HEU children having twice as many transversions in the D-loop than unexposed children [28]. However, mtDNA deletions have rarely been investigated, and no study has reported their exhaustive cartography so far.

## 2. Materials and Methods

### 2.1. Study Population

We analyzed 32 HEU children at seven days of life who were previously enrolled in the PROMISE PEP trial (NCT00640263). The trial was conducted between November 2009 and May 2012 in Burkina Faso, South Africa, Uganda and Zambia, and it enrolled mother–child pairs for a safety and efficacy evaluation of an infant prophylaxis given from seven days after birth to one year of age to prevent HIV transmission through breastfeeding [29]. HIV-infected mothers were not eligible for an ARV treatment at the time of the study but received ARV prophylaxis during pregnancy consisting of zidovudine (ZDV) alone or combined with lamivudine. It is noteworthy that in the present study, all 32 mothers of the HEU children were among those who received ZDV only. Children received 6 days of nevirapine (NVP) from birth. All demographic and anthropometric data were obtained from the PROMISE-PEP trial database.

We also analyzed anonymously 15 HIV-unexposed uninfected neonates collected in France for the systematic screening of inherited diseases at birth in 2019 (*n* = 9) and in 2012 (*n* = 6) to check the absence of bias due to DNA alteration with time on dried blood spots (DBS). Of note, 40.0% of these latter controls carried an mtDNA African haplogroup to check the bias due to the geographic origin of participants.

### 2.2. Sample Collection and Processing

Whole blood was collected by heel prick seven days after birth directly processed on DBS (Whatman 903 cards) stored with desiccant in a zipped pouch at −20 °C at the study sites. DNA was extracted using the QIAamp DNA Blood Mini Kit (Qiagen, Hilden, Germany), following the manufacturers’ instructions, from 3 mm diameter punches (*n* = 3) and stored at −80 °C.

Whole blood was also collected by venipuncture. Peripheral blood mononuclear cells were isolated from whole blood by Ficoll density gradient centrifugation. Dried cell pellets were cryopreserved at −80 °C for subsequent testing.

### 2.3. Detection and Quantification of Mitochondrial DNA Deletion: The eKLIPse High-Throughput Computational Pipeline

The mtDNA deletions and point mutations were assayed by an assay combining deep high-throughput sequencing of mtDNA and a dedicated bioinformatic pipeline as described in [30]. Briefly, mtDNA (NC_012920.1) from DBS DNA extracts was amplified in two overlapping fragments of 8009 bp and 8994 bp using the following forward and reverse primer 5’-TACGTTGTAGCCCACTTCCACT-3’ and 5’-GCCCGATGTGTAGGAAGAG-3’, and 5’-AACTTCGGCTCACTCCTTGG-3’ and 5’-AGTAACGTCGGGGCATTCCG-3’ and the long-range LA Taq DNA polymerase (Takara Bio Europe SAS, Saint-Germain-en-Laye, France). The PCR was performed as follows: 94 °C 1 min, (98 °C 20 s, 60 °C 3 min, 72 °C 12 min) 35 times, 72 °C 10 min, 4 °C until use. To verify the absence of mtDNA pseudogenes amplification, which is present in the nuclear genome, the absence of amplification was first tested on DNA extracted from Rho zero cells, which are known to be devoid of mtDNA. Moreover, a negative control was included in all batches and followed the same workflow as all samples to check for the absence of contaminations. Next, amplified mtDNAs were fragmented using an enzymatic fragmentation approach, and adaptors and barcodes sequences were added to the DNA fragments using the AB Library Builder system (Ion Xpress™ Plus Fragment Library Kit, Thermo Fisher Scientific, Waltham, MA, USA). Then, emulsion PCR was performed using an Ion Chef apparatus (Thermo Fisher Scientific, Waltham, MA, USA), and DNA fragments were sequenced on the Ion S5-XL on an Ion 540 chips (Thermo Fisher Scientific, Waltham, MA, USA). After bioinformatics processing, various quality controls were checked: run parameters meeting the manufacturer recommendations, number of mapped reads, minimal sequencing depth of 100 reads per base, no contamination with the negative control sample. eKLIPse provides the precise breakpoint positions and the cumulative percentage of mtDNA rearrangements at a given gene location. For this study, we determine the lower limit of detection for each mtDNA position by the detection of at least ten reads out of the total reads, given that none of the control samples presented deleted mtDNAs in our routine practice. As a consequence, the heteroplasmy rate may be lower than the one previously published [30]. This threshold of quantification was set for diagnostic purposes and in a rather conservative manner. However, in the context of a research study, events of lower frequency may be indicative of a subclinical genetic instability. Therefore, the heteroplasmy rate from 0.01% to 0.5% will be not accurately quantified but is indicative of an mtDNA instability. To ensure the reproducibility of the mtDNA deletion detection method, we applied the entire workflow (long-range PCRs, library preparation, PCR emulsion, sequencing, and bioinformatics analysis) two times on 22 out of the 32 HEU samples. Analyses confirmed that the mtDNA deletion detection is robust and reproducible (unplublished material). The mtDNA deletions were evidenced in total DNAs extracted from dried blood spots, but the same rates of deletion were observed with total DNAs extracted from dried cell pellets. We also checked the absence of mtDNA deletions in control neonates with the same DBS duration of conservation (8 years) to avoid biased results due to DNA alteration. The cartography of mtDNA deletion is given by a graphical interface through a mitocircle representation. Mitochondrial DNA variants were detected, annotated, and prioritized as described previously [31]. In short, next-generation sequencing reads were analyzed with a dedicated in-house pipeline for coverage analysis, variant calling, annotation, and prioritization. Then, the identified variants were analyzed individually to determine biological relevance using a funnel strategy with the following criteria: (i) status in Mitomap database, (ii) frequency in mtDNA variants databases (Genbank, GnomAD and Hellix), (iii) conservation percentage of the nucleotide, and (iv) classification of the variant by in silico pathogenicity predictor tools (MitImpact, APOGEE, SIFT, and POLYPHEN).

### 2.4. Statistical Analysis

The participant characteristics were described using means with standard deviations (SD) or medians with interquartile range (IQR) for continuous variables as well as percentages for categorical variables.

We investigated the association between having mtDNA deletions at day seven and the following explanatory variables, using Poisson regressions with robust error variance: sex, gestational age, weight of the child at day seven, study site, age of the mother, maternal viral load at D7, duration of ZDV prophylaxis taken during pregnancy and alcohol consumption during pregnancy. To investigate whether these variables could also influence the level of heteroplasmy, we dichotomized the cumulative heteroplasmy rate, setting the threshold at 0.5%, and performed the same analysis.

Statistical analyses were performed using SAS studio (Copyright © 2012–2016, SAS Institute Inc., Cary, NC, USA).

## 3. Results

### 3.1. Study Population

Nineteen (59.4%) of the HEU children in our study originate from Burkina Faso. The sex ratio was close to 1:1, but it was 2:1 (boy:girl) in the control group. Their growth indicators were in the normal range according to WHO standards and did not differ from the values of the other children enrolled in the trial when matched by country (data not shown). During pregnancy, all mothers received ZDV as prophylaxis, but one-third had a HIV viral load above 1000 copies/mL at day 7 post-partum. None of them have smoked during pregnancy, but half of them drank alcohol (Table 1). Children of the control group were of different origins, but 40.0% shared the same haplogroups L of African origin (see Supplementary File 1).

### 3.2. Deletion Profile among HEU Children

**Figure 1:**
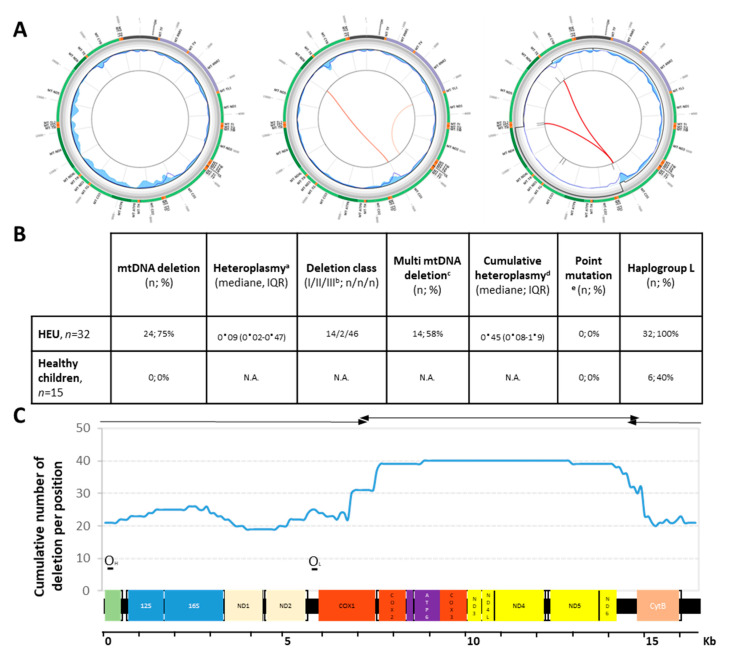
mtDNA deletions among HEU children. (**A**) Schematic representation of the eKLIPse high-throughput computational pipeline result. The outer circle represents the mtDNA genome. Deletions are visualized in the center of the circles as red lines. Left: a subject with no deletion, middle: a subject with two deletions of different heteroplasmy, right: a subject with two deletions of high heteroplasmy. The width of the red line varies according to the percentage of heteroplasmy. The inner circle contains data related to the sequencing coverage and quality control of the sequencing (see [30] for details). (**B**) The data for each child population. a: heteroplasmy corresponds to the percentage of deleted to wild-type mtDNA molecules; b: class I deletions are flanked by perfect repeats, class II deletions are flanked by imperfect repeats, and class III deletions are not flanked by any similar sequences; c: multiple mtDNA deletions are defined as having more than one type of deletion, d: cumulative heteroplasmy is defined as the sum of each heteroplasmy for one individual; e: point mutation is defined as any heteroplasmic pathogenic variant. IQR: first and third interquartile range; HEU: HIV-exposed uninfected children. (**C**) Deletion profile among HEU children. The cumulative number of deletions per position was plotted against a linear mitochondrial genome. Products of long-range PCR amplification are depicted on top of the diagram. The two origins of replication (OH and OL) are located.

After applying rigorous quality control procedures for NGS [30], we observed that 24/32 HEU children (75.0%) carried significant deletions in mtDNA, irrespective of the country of origin, 41.7% carrying a single deletion, and 58.3% carried multiple deletions (Figure 1B). The heteroplasmy rates, i.e., the coexistence of deleted and wild-type mtDNA within cells, ranged from 0.01% up to 50.2%, with a cumulative median of 0.45% (IQR: 0.08–1.9, full description in Supplementary File 1). Out of them, 16 (25%) were in the linear range of quantification. Deletions were flanked by two direct repeats with identical sequences (class I) in 14 cases (22.5%), by imperfect repeats (class II) in 2 cases (3.2%), and by without direct repeats (class III) in 46 cases (74.2%). Among these deletions, 33 (53.2%) conserved the two origins of replication, 10 (16.1%) lost one origin, and 19 (30.6%) lost both. Most of the deletions were about half of the mitochondrial genome (∆8000 bp) and spanned within the major Arc region (37/62 (59.7%), Figure 1A). In contrast, heteroplasmic pathogenic single nucleotide sequence variants were never observed in these children, and if observed, they were most likely polymorphism. In the control group of children born to HIV-uninfected mothers (*n* = 15), neither deletion nor point mutations were detected at birth (full description in Supplementary File 1).

### 3.3. Predictive Factors for mtDNA Instability

None of the following factors were predictive for “having a mtDNA deletion” or “having a cumulative heteroplasmy rate higher than 0.5%”: “sex of the child”, “gestational age”, “weight of the child at day seven”, “study site”, “duration of ZDV prophylaxis taken during pregnancy”, and “alcohol consumption during pregnancy”, but “mother viral load at day seven” was predictive for both the presence of deletion and heteroplasmy level (PR = 1.27; 95%CI: 1.01–1.58, *p* = 0.03 and PR = 1.54; 95%CI: 1.02–2.34, *p* = 0.04, respectively), suggesting a possible direct impact of HIV or HIV proteins on mtDNA instability. “Being born of a mother older than 30 years” was also identified as a risk factor for “having a mtDNA deletion” (PR = 1.46; 95%CI: 1.00–2.13, *p* = 0.05) (Table 2).

## 4. Discussion

Our study reveals that mtDNA integrity is commonly affected in HEU neonates. Since blood cells have a high division rate allowing the rapid elimination of altered mtDNA deletions that rather preferentially accumulate with age in post-mitotic tissues such as brain or skeletal muscles [32], the proportion of mtDNA deletion in HEU children may be higher in these organs than in blood. Indeed, such levels of deletions are rarely encountered in clinical practice of inherited mitochondrial disorders in blood. For example, deletions well known to accumulate with age in post-mitotic tissues are in fact rarely observed in the blood of patients, including the elderly [33].

The time of acquisition of these deletions is uncertain. mtDNA deletions are generally sporadic and almost never inherited, their transmission being prevented by the bottleneck mitochondrial selection during early oogenesis, which ensures the counter selection of mutant [34]. We suggest that these mtDNA deletions occur de novo later during the maternal germline (HIV exposure) or embryo (HIV and ARV prophylaxis exposures) developments.

Class I deletions are driven by DNA–polymerase–gamma defaults, while class III deletions, found in 74% of cases in our study, are more frequently associated with the aging process [35,36]. Furthermore, half of the deletions encompassed one or the two origins of replication, which questions how these molecules replicate and thus could be detected. It is noteworthy that our NGS approach uses information from short DNA fragments (median 180 bp) sequencing and does not provide information on the full structure of mtDNA. The observed deletions are likely to originate from an incomplete duplication of mtDNA resulting in a chimeric molecule, the full-length sequence conferring the replication origins lacking in the deleted sequence, as already hypothesized by others [37]. Such rearrangements have already been described in human tumors and the aging process [35,36,38].

The children were exposed both to maternal and infant ARV prophylaxis; mothers received ZDV during pregnancy and a single dose of NVP during labor, and children received NVP for their first 6 days of life. mDNA instability was assessed before the initiation of the study drugs (at day 7) in the PROMISE-PEP trail, preventing any interaction other than with drugs used routinely for the prevention of mother-to-child transmission (MTCT) of HIV [29]. Even if ZDV and NVP are known to be mitotoxic, only one pharmaco-toxicologic study has reported mtDNA deletions in murine cortical neurons upon administration of ARVs, including ZDV [39]. No reported studies investigated the effect of various ARVs on the occurrence of mtDNA deletions in humans. Given the new guidelines and practices, HIV-infected pregnant women are now receiving triple ARV therapy, and it is now recommended that HEU children receive an extended prophylaxis up to the end of breastfeeding in order to tackle MTCT of HIV [40,41]. Such intensification of ARV exposure, up to triple-drug prophylaxis for the child, as well as the prolonged exposure to more than one year strongly advocate for a full assessment of mtDNA integrity and long term clinical follow up.

The children were also exposed to maternal HIV and HIV proteins. HIV-induced mitochondrial dysfunctions were described among HIV-infected people, and it was suggested that HIV can hijack mitochondria with consequences such as enhanced viral transmission and inhibited immune response [42]. Various mechanisms have been described in vitro, including an inhibition of the mitophagy process, which results in an accumulation of “non-functional” mitochondria [43]. If mitochondria dysfunctions are also impaired among HEU children, it was never attributed to maternal-transmitted viral proteins. Herein, mother with higher viral load was a risk factor for detecting mtDNA deletion, suggesting that this hypothesis is plausible and requires further investigations.

The clinical expression of the mtDNA deletions is related to a threshold effect depending on the heteroplasmy rate which evolves with age, on the stochastic tissue distribution of deleted mtDNA and on the unequal vulnerability of affected tissues to impaired oxidative metabolism [34]. Even very low rates of deletions detected in blood by deep sequencing may reveal mtDNA maintenance disorders with potential severe clinical consequences. Future steps will require further investigation of deleted mtDNA molecules later in HEU children’s lives in both qualitative and quantitative terms. Mechanisms of elimination exist and are efficient up to a certain threshold of heteroplasmy [44]. However, should they become inefficient, mtDNA deletions observed herein are likely to contribute, at least partially, to the health impairments observed in HEU children, especially those with increased deletion contents. It is striking that cardiac conduction dysfunctions, muscle weakness, or elevated lactate concentrations reported in HEU children [45,46,47] are also hallmarks of most mitochondrial disorders.

This study has several limitations. Firstly, it is a post hoc analysis of samples collected for the PROMISE-PEP trial. As a consequence, the control group was constructed retrospectively. We did not have access to country-matched controls; however, we did our best to include controls with the L haplogroup and the same date of collection. Secondly, the number of samples included in the analysis is small. Thirdly, control samples were collected on average 4 days before those of the PROMISE trial. To the best of our knowledge, it is unlikely that the time frame for the acquisition of mtDNA deletion will occur specifically during these 4 days. Fourthly, as PMTCT recommendations have evolved, the population analyzed herein is not representative of the current HEU but can be applied to all HEU born before 2013. Fifthly, the study is purely descriptive and cannot predict any causality for future health outcomes for these children. Finally, we used a minimal number of reads with a breakpoint of 10 to include the deletion in the analysis. The use of this threshold allows for the description of low-frequency events and renders possible the reporting of low-level genetic instability. Control samples and routine practice clearly demonstrate the success of this threshold. As this is an inference, we may underreport these deletion events for samples with low sequencing depth.

## 5. Conclusions

The consequence and the evolution over time of such mtDNA instability occurring early in life is presently unknown. Long-term clinical follow up of children at high risk of mitochondrial defects is mandatory. The benefit of using of other drugs potentially toxic to mitochondria such as chloramphenicol, ethambutol, linezolide, or valproic acid [48] should also be carefully weighted in this context of iatrogenic mito-senescence.

## Figures and Tables

**Table 1:** Characteristics of the mother–child pair at day seven post-partum.

Children	*n* = 32
Sociodemographic	
Site; *n* (%)	
Burkina Faso	19 (59.4)
South Africa	4 (12.5)
Zambia	9 (28.1)
Gender; *n* (%)	
Boy	17 (53.1)
Anthropometry	
Weight (kg); mean ± SD	3.1 ± 0.4
Height (cm); mean ± SD	49.1 ± 2.0
WAZ; mean ± SD	−0.7 ± 0.8
HAZ; mean ± SD	−0.9 ± 1.0
WHZ; mean ± SD	−0.4 ± 1.2 ^†^
Gestational age (week); median [IQR]	38.0 [37.0;39.0]
Preterm birth (week); *n* (%)	
No prematurity ≥ 37	28 (87.5)
Prematurity < 37	4 (12.5)
Hematology	
Hemoglobin (g/dL); mean ± SD	15.6 ± 2.0
Hemoglobin (g/dL); *n* (%)	
Normal > 13	28 (87.5)
Anemia ≤ 13	4 (12.5)
Mild [12;13]	4 (12.5)
Platelet count (10^3^/mm^3^); *n* (%)	
Normal ≥ 125	32 (100.0)
White cell count (10^3^/mm^3^); *n* (%)	
Normal > 2.5	32 (100.0)
Neutrophil count (10^3^/mm^3^); *n* (%)	
Normal > 1.5	30 (93.8) ^†^
Neutropenia ≤ 1.5	1 (3.2) ^†^
Mild [1.25;1.5]	1 (3.2) ^†^
Biochemistry	
ALAT (U/L); *n* (%)	
Normal ≤ 40	31 (96.9)
Abnormal > 40	1 (3.1)
Mild [40;100]	1 (3.1)
**Mother**	***n* = 32**
Sociodemographic characteristics	
Age (year); mean ± SD	29.0 ± 5.3
Parity; median [IQR]	3.0 [2.0;3.5]
Education	
Mother/caregiver ever attended school; *n* (%)	
Yes	23 (82.1) ^‡^
Clinical and biological characteristics	
BMI; median [IQR]	23.5 [21.6;26.2]
CD4 cells count (cells/mm^3^); median [IQR]	532.5 [432.5;734.0]
HIV viral load control (copies/mL); *n* (%)	
<1000	20 (62.5)
≥1000	12 (37.5)
WHO HIV staging; *n* (%)	
Stage 1	32 (100.0)
Maternal prophylaxis during pregnancy	
ARV regimen; *n* (%)	
ZDV	32 (100.0)
Duration of ARV prophylaxis (week); median [IQR]	8.8 [5.0;10.5]
Maternal lifestyle during pregnancy	
Smoking during pregnancy; *n* (%)	
No	24 (100.0) ^§^
Alcohol consumption during pregnancy; *n* (%)	
Yes	12 (50.0) ^§^

^†^ one missing value, ^‡^ four missing values; ^§^ eight missing values. Abbreviations: SD, standard deviation; IQR, interquartile range; WAZ, weight-for-age Z-score, HAZ, height-for-age Z-score; WHZ, weight-for-height Z-score; BMI, body mass index; HIV, human immunodeficiency virus; WHO, World Health Organization; ARV, antiretroviral; ZDV, zidovudine.

**Table 2:** Predictive factors for mitochondrial instability.

	Deletion Yes vs No (*n* = 32)			Cumulative Heteroplasmy Rate ≥ 0.5 (*n* = 32)		
	PR (95%CI)		*p* Value	PR (95%CI)		*p* Value
Site (ref. = Zambia)						
Burkina Faso	1.61 (0.88–2.95)		0.12			
South Africa	0.90 (0.29–2.82)		0.86			
Sex (ref. = Female)						
Male	1.04 (0.70–1.56)		0.84	0.63 (0.25–1.57)		0.32
Age of the mother (per one year)	1.03 (0.99–1.07)		0.16	1.03 (0.95–1.12)		0.47
Age of the mother (ref. ≤ 30 years						
≥30 years	1.46 (1.00–2.13)		0.05	2.05 (0.83–5.01)		0.12
Mother viral load at D7 (Log copies/mL)	1.27 (1.01–1.58)		0.03	1.54 (1.02–2.34)		0.04
Duration of maternal prophylaxis (per week)	0.99 (0.95–1.04)		0.77	0.95 (0.87–1.04)		0.27
Gestational age (per week)	1.02 (0.95–1.10)		0.54	0.89 (0.69–1.15)		0.37
Weight of the child at D7 (per 500 g)	1.07 (0.85–1.34)		0.59	0.92 (0.49–1.75)		0.81
Alcohol consumption during pregnancy (ref. = No)	0.89 (0.53–1.49)		0.65	1.00 (0.39–2.58)		1.00

PR: prevalence ratio; CI: confidence interval.

## Data Availability

The data presented in this study are available in Supplementary Materials here.

## Supplementary Materials

The following are available online at https://www.mdpi.com/article/10.3390/jcm10112399/s1, Table S1: Full data set.
